# Monitoring therapeutic efficacy of sunitinib using [^18^F]FDG and [^18^F]FMISO PET in an immunocompetent model of luminal B (HER2-positive)-type mammary carcinoma

**DOI:** 10.1186/s12885-015-1540-2

**Published:** 2015-07-22

**Authors:** Benoît Thézé, Nicholas Bernards, Audrey Beynel, Stephan Bouet, Bertrand Kuhnast, Irène Buvat, Bertrand Tavitian, Raphaël Boisgard

**Affiliations:** 1 Laboratoire Imagerie Moléculaire In Vivo (IMIV, UMR 1023 Inserm/CEA/Université Paris Sud - ERL 9218 CNRS, CEA/I²BM/SHFJ, 4 place du Général Leclerc, 91400 Orsay, France; 2 Animal Genetics and Integrative Biology, INRA-AgroParisTech, UMR 1313, Jouy-en-Josas, France; 3 Laboratory of Radiobiology and Genomics Studies, CEA, DSV, IRCM, SREIT, Jouy-en-Josas, France; 4 Inserm U970, Université Paris Descartes, Paris, France

**Keywords:** Breast cancer, PyMT, Sunitinib, PET, Digital microscopy

## Abstract

**Background:**

Clinical studies implying the sunitinib multi-kinase inhibitor have led to disappointing results for breast cancer care but mostly focused on HER2-negative subtypes. Preclinical researches involving this drug mostly concern Triple Negative Breast Cancer (TNBC) murine models. Here, we explored the therapeutic efficacy of sunitinib on a PyMT-derived transplanted model classified as luminal B (HER2-positive) and monitored the response to treatment using both *in vivo* and *ex vivo* approaches.

**Methods:**

Tumour-induced animals were treated for 9 (*n* = 7) or 14 (*n* = 8) days with sunitinib at 40 mg/kg or with vehicle only. Response to therapy was assessed *in vivo* by monitoring glucose tumour metabolism and hypoxia using 2-deoxy-2-[^18^F]fluoro-D-glucose ([^18^F]FDG) and [^18^F]fluoromisonidazole ([^18^F]FMISO) Positron Emission Tomography (PET). After primary tumour excision, e*x vivo* digital microscopy was performed on treated and control samples to estimate vascular density (CD31), apoptosis (Tunel), proliferation (Ki-67), Tumour-Associated Macrophage (TAM) infiltration (F4/80), metabolism (GLUT1) and cellular response to hypoxia (HIF1 alpha). The drug impact on the metastasis rate was evaluated by monitoring the PyMT gene expression in the lungs of the treated and control groups.

**Results:**

Concomitant with sunitinib-induced tumour size regression, [^18^F]FDG PET imaging showed a stable glycolysis-related metabolism inside tumours undergoing treatment compared to an increased metabolism in untreated tumours, resulting at treatment end in 1.5 less [^18^F]FDG uptake in treated (*n* = 4) vs control (*n* = 3) tumours (*p* < 0.05). With this small sample, [^18^F]FMISO PET showed a non-significant decrease of hypoxia in treated vs control tumours. The drug triggered a 4.9 fold vascular volume regression (*p* < 0.05), as well as a 17.7 fold induction of tumour cell apoptosis (*p* < 0.001). The hypoxia induced factor 1 alpha (HIF1 alpha) expression was twice lower in the treated group than in the control group (*p* < 0.05). Moreover, the occurrence of lung metastases was not reduced by the drug.

**Conclusions:**

[^18^F]FDG and [^18^F]FMISO PET were relevant approaches to study the response to sunitinib in this luminal B (HER2-positive) model. The sunitinib-induced vascular network shrinkage did not significantly increase tumour hypoxia, suggesting that tumour regression was mainly due to the pro-apoptotic properties of the drug. Sunitinib did not inhibit the metastatic process in this PyMT transplanted model.

**Electronic supplementary material:**

The online version of this article (doi:10.1186/s12885-015-1540-2) contains supplementary material, which is available to authorized users.

## Background

Based on encouraging preclinical data, the sunitinib drug, a multi-kinase inhibitor, has been investigated in several clinical studies in association with various cytotoxic drugs but led to disappointing results in breast cancer patients. Classically, breast cancers are classified according to the expression levels of the estrogen (ER) and progesterone (PR) receptors, and of the human epidermal growth factor receptor 2 (HER2) oncogene. The large majority of studies with sunitinib involved advanced and heavily treated breast cancer focusing on the HER2-negative (or trastuzumab (TZM) insensitive) subtypes (phase I [1], phases II [2, 3], phases III [4–6]). Interestingly, Burstein et al. administered sunitinib alone and detected superior overall response rate (ORR) for the HER2-positive subtype (25 % vs 11 % in the whole population) [7]. Moreover, two recent reports focusing on HER2-positive breast cancers found an improved ORR in adding sunitinib to regimens based on TZM with or without docetaxel administration [8, 9].

The breast cancer classification is now reconsidered in the light of global gene expression analyses of human biopsies leading to six identified subtypes: luminal A and B, basal-like, claudin-low, HER2-enriched and normal breast like [10, 11]. A detailed panorama of the relationships between the histological- and the transcriptomic-based classifications has been recently published [12]. In this context, the breast cancer patient care is evolving as it is expected that the efficacies of chemotherapeutic regimens should depend on the considered subtype.

In the case of sunitinib, many preclinical studies were performed using various Triple Negative Breast Cancer (TNBC) mouse models, and all found that the drug delayed the tumour growth at doses ranging between 20 to 60 mg/kg/day. Interestingly, sunitinib treatment induced tumour regression in a MCF7 xenograft model [13], which is a typical luminal A cancer [14], as well as on a MMTV-v-Ha-Ras transgenic model [15], which has been classified as luminal B [16]. Among the breast cancer diversity, the luminal subset represents mainly the ER+ group, for which an endocrine therapy is recommended. The luminal A cancers are defined as ER+ PR+ HER2- and low Ki-67 whereas luminal B carcinomas are ER+ HER2+ or ER+ PR+/− HER2- and high Ki-67 [17, 18]. The luminal A cancers present a relatively good outcome, but the luminal B tumours, which represent 10 to 20 % of all breast cancers, are associated with a poor prognosis and identification of new therapeutic options for this subtype is still very challenging. Thus, as most anterior preclinical studies with sunitinib focused on TNBC models, we investigated here its efficacy in a luminal B-type breast cancer model combining *in vivo* PET and *ex vivo* histochemical analyses of tumours.

For this purpose, we used the MMTV-PyMT murine model whose oncogenesis is induced by expression of the polyoma virus middle T oncoprotein under control of the Mouse Mammary Tumour Virus (MMTV) promoter (PMID: 1312220). Following the recommendations of Varticovski et al. [19] about the limitations of using genetically engineered mouse models in preclinical studies, we generated a transplanted orthotopic and syngeneic model from the original transgenic mice. In order to characterize the therapy response to sunitinib in the PyMT model, we then performed *in vivo* Positron Emission Tomography (PET) with 2-deoxy-2-[^18^F]fluoro-D-glucose ([^18^F]FDG) and [^18^F]fluoromisonidazole ([^18^F]FMISO) radiotracers, which allow to monitor tumour glucose metabolism and hypoxia respectively. Furthermore, *in vitro* analyses were used to quantify the chemotherapy impact on several cancer-associated parameters, namely vascularization (CD31), apoptosis (TUNEL), proliferation (Ki-67), hypoxia (HIF1 alpha), TAM infiltration (F4/80), metabolic activity (GLUT1) and metastasis.

## Methods

Animal studies were approved by the animal ethics committee “Comité d'EThique en Expérimentation Animale” (CETEA DSV n°44) under reference 12–036 and conducted in accordance with the Directives of the European Union.

### Tumour removal and preparation of cell suspensions

FVB/N-Tg (MMTV-PyMT)634Mul/J (PyMT) 12-weeks-old mice were used as tumour donor. Aseptically collected mammary tumours from PyMT mice were minced and immersed in cold Dulbecco's Modified Eagle's Medium (Sigma, USA). Mechanical cell dissociation was performed using Medicon disposable chambers (BD bioscience, USA). The cell suspension was then progressively filtered using Filcon filters with pore sizes of 500 μm, 200 μm and 70 μm (BD bioscience). Finally, cells were aliquoted in freezing medium (Life Technologies, USA) and stored in liquid nitrogen.

### Tumour implantation and monitoring

After freezing medium removal and enumeration, the tumour cells were directly inoculated, without any *in vitro* culture step, in the mammary fat pad of the posterior nipple in FVB mice. The tumour volumes were calculated using calliper measurements and the approximated formula for a prolate ellipsoid, given by:

$$ \mathrm{Volume}\ \left(\mathrm{m}{\mathrm{m}}^3\right) = \left(\mathrm{Length}\ \left(\mathrm{m}\mathrm{m}\right) \times \mathrm{Widt}{\mathrm{h}}^2\left(\mathrm{m}{\mathrm{m}}^2\right)\right)\ /2. $$

To evaluate drug toxicity, body animal weights were also monitored.

### Chemotherapy

Two sets of mice were used in this study. For the main set A, 7 animals were implanted with 3 million viable cells. PyMT tumours were allowed to grow for 21 days. The mice were randomized into treated (*n* = 4) and control (*n* = 3) groups. The treated one received per os a daily dose of sunitinib at 40 mg/kg in 20 mM dimethyl sulfoxyde (DMSO, Sigma). The control group received only the DMSO solution. Drug administration was performed during 9 consecutive days.

To further explore the neoadjuvant therapy effects on the metastatic incidence, we extended primary tumour growth and treatment times before mammary tumour surgical resection. Thus, a secondary set B of treated (*n* = 4) and control (*n* = 4) mice was obtained implanting 400 000 viable cells. Treatment began at day 25 post implantation. It continued for 14 days until resection at day 39. For both sets A and B, the primary tumours were surgically removed after treatment and the mice were kept alive for 60 supplementary days before euthanasia to analyse the lungs for metastasis content.

### [^18^F]FDG and [^18^F]FMISO positron emission tomography

[^18^F]FDG and [^18^F]FMISO PET scans were performed on mice from set A at days 0 and −1 respectively prior to treatment and at days 5 and 6 of treatment. 15 min long PET acquisitions were performed 60 min after [^18^F]FDG injection and 90 min after [^18^F]FMISO injection. PET data were corrected for attenuation, scatter and radioactive decay and reconstructed using a two dimensional ordered-subset expectation maximization (2D-OSEM) algorithm after Fourier rebinning, with a voxel size of 0.5 × 0.5 × 0.8 mm^3^ (sofware ASIPro VM™, CTI Concorde Microsystems). Radioactivity uptake in regions of interest (ROIs) was measured using BrainVISA 4.0 and Anatomist 4.0.2 (CEA/Neurospin/SHFJ, France) and expressed in Standardized Uptake Value (SUV) calculated using:

$$ \mathrm{S}\mathrm{U}\mathrm{V} = \left[\mathrm{percent}\ \mathrm{of}\ \mathrm{injected}\ \mathrm{dose}\ \mathrm{per}\ \mathrm{gram}\ \left(\%\mathrm{ID}/\mathrm{g}\right) \times \mathrm{body}\ \mathrm{mass}\ \left(\mathrm{g}\right)\right]/100. $$

### Histochemistry

Primary tumours from set A of animals were fixed in zinc solution (BD bioscience) and included in paraffin. Series of tissue sections were sequentially cut. For blood vessels, macrophages and cellular hypoxia sensor labelling, the slides were immersed in toluene and progressively rehydrated. Endogenous peroxidases and biotin were blocked with 3 % hydrogen peroxide solution (Sigma) and biotin blocking kit (Life technologies) respectively. Rat anti CD31 (Pharmingen, USA), rat anti F4/80 (Caltag, UK) and rabbit anti Hypoxia Inducible Factor 1 alpha (HIF1 alpha, LSBio, USA) were used as primary antibodies for each labelling respectively. Biotin-goat anti rat IgG (Life technologies) was used as secondary antibody for vascular and macrophage staining. The tyramide signal amplification (TSA) system (Perkin Elmer, USA) was then used following manufacturer’s instructions. For HIF1 alpha labelling, HRP-goat anti rabbit IgG (Life technologies) was incubated as secondary antibody. For cellular proliferation and Glucose transporter 1 (GLUT1) expression labelling, paraffin removal was performed using heated PT module buffer pH8 (Fischer Scientific, USA). As above, after the blocking steps, the slides were incubated with goat anti Ki-67 (Santa Cruz, USA) or rabbit anti GLUT1 (Neomarker, USA) for each labelling respectively. Secondary detection reagents were biotin-rabbit anti goat IgG (Life technologies) followed by TSA system for Ki-67 or HRP-goat anti rabbit IgG (Life technologies) for GLUT1. After 3-3'–diamino-benzidine (DAB, Sigma) revelation, counterstaining was performed with hematoxylin (Sigma) and slides were mounted with Eukitt (Sigma). For late apoptosis staining, terminal deoxynucleotidyl transferase dUTP nick end labelling (TUNEL, Promega, USA) was used according to the manufacturer’s protocol. Slides were then mounted with ProLong Gold Antifade Reagent containing 4',6'-diamidino-2-phenylindole (DAPI, Life technologies).

### Microscopy image acquisition and analysis methods

The set of tissue sections uniformly sampling the whole volume of each tumour was entirely scanned at high resolution (0.37 μm per pixel) using an AxiObserver Z1 (Zeiss, Germany). The resulting brightfield image series were analysed using the CellProfiler software [20]. After a colour deconvolution step, the segmentation of each structure of interest was based on a constant labelling-dependent threshold. A filtering step was added for size-based vessel clustering. Logic diagrams of the processing pipelines are available as supplementary data (see Additional files 1 and 2). The DAB-labelled surface areas and whole hematoxylin areas were measured by the software. The consistency of the automatic segmentation was controlled visually on the original images supplemented with the outlines of identified objects. Whole tissue sections fluorescently labelled with the TUNEL method were acquired using two excitation/emission filter sets: 365/445 nm for DAPI and 470/525 nm for TUNEL staining. TIF-format images were processed using the ImageJ software [21], yielding the total area corresponding to fluorescent pixels above a given constant threshold. The measured areas were multiplied by the distance between each tissue slide to get volume estimates.

### Quantitative real time polymerase chain reaction (qRT-PCR)

The whole-lung tissue ribonucleic acids (RNA) were extracted using the total RNA isolation kit (Macherey-Nagel, Germany) following manufacturer’s instructions. RNA was reverse transcribed using SuperScript II (Life technologies) with random primer hexamers. On a LightCycler 1.5 (Roche, Switzerland), a subsequence of the PyMT cDNA was amplified in Master SYBR Green I mix (Roche) using the previously described primers [22]. The housekeeping myelin protein zero (P0, MPZ) gene was used as an internal control. A relative quantification analysis was performed applying the delta-delta Ct method.

### Statistical analyses

For statistical analysis, unpaired Student t-tests were performed using GraphPad Prism software. A p-value of 0.05 or less was interpreted as statistically significant. In all graphs, values are reported as mean ± one standard deviation (SD).

## Results

### Sunitinib-induced mammary tumour regression on the PyMT model

**Fig. 1:**
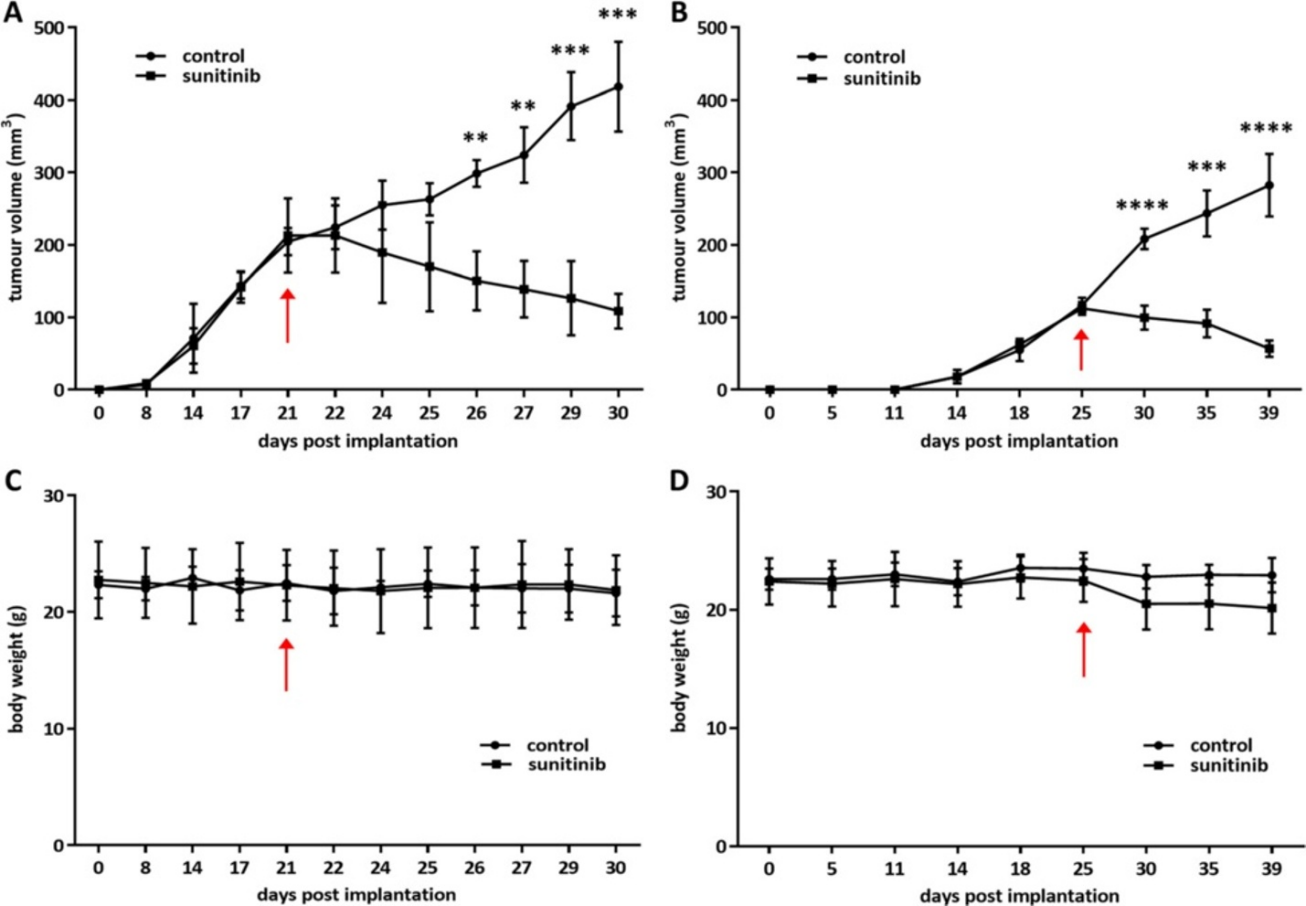
*In vivo* therapy model follow-up. **a**-**b** Tumour volume evolution, as measured by calliper, for sets A and B of mice respectively (set A: *n* = 3 for control, *n* = 4 for treated/set B: *n* = 4 for each group). **c**-**d** Mice body weight evolution for sets A and B respectively. In all graphs, arrows indicate the first day of sunitinib treatment

In set A of mice, the mean tumour volume measured by calliper was 209 ± 38 mm^3^ (*n* = 7) just before treatment (day 21). During the treatment phase until day 30, the tumours of the control group continued to grow up to 418 ± 62 mm^3^ (*n* = 3), while the size of the treated tumours decreased down to 109 ± 24 mm^3^ (*n* = 4) (Fig. 1a, *p* < 0.001). In set B of mice, the mean tumour volume measured by calliper was 115 ± 9 mm^3^ when treatment started (day 25, *n* = 8). At resection (day 39), tumour volumes were 282 ± 43 mm^3^ in the control group (*n* = 4) and 57 ± 11 mm^3^ in the treated group (*n* = 4) (Fig. 1b, *p* < 0.0001). The mouse weights corrected for their tumour weight (Fig. 1c-d) were not significantly different between the treated and control arms.

### Effect of sunitinib administration on [^18^F]FDG and [^18^F]FMISO *in vivo* uptakes

**Fig. 2:**
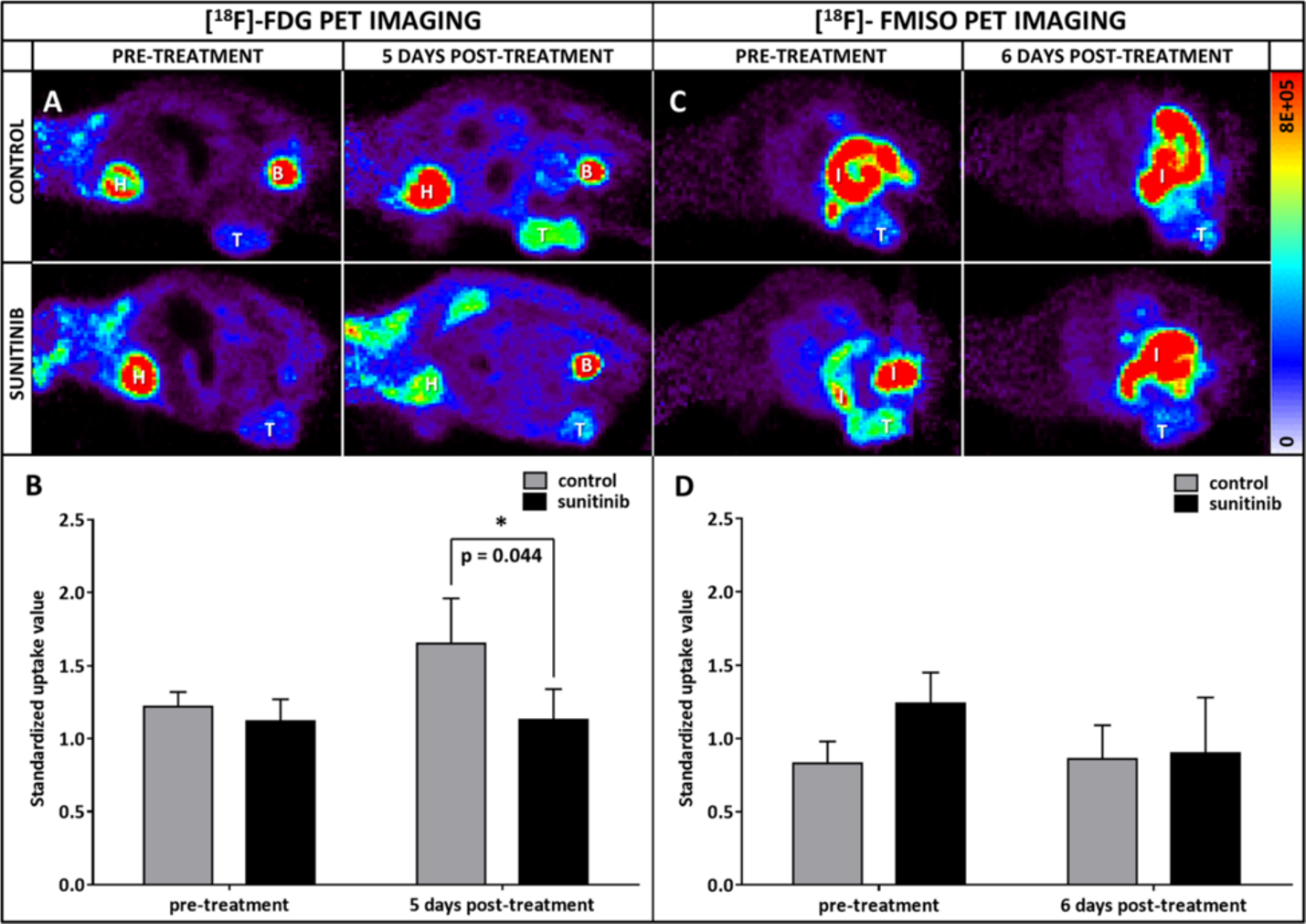
Evolution of PET radiotracer uptakes. **a** Representative images of a tumour-bearing mouse injected with [^18^F]FDG prior to treatment (left) and after 5 days (right) of sunitinib (lower part) or DMSO (upper part) administration (B: bladder, H: heart, T: tumour). **b** Tumour [^18^F]FDG SUV evolution for both 5 day-treated (*n* = 4) and control (*n* = 3) groups. **c** PET longitudinal images of a grafted mice injected with [^18^F]FMISO before treatment (left) and after 6 days (right) of treatment with sunitinib (bottom) or DMSO only (top) (I: intestine, T: tumour). **d** Tumour [^18^F]FMISO uptake evolution for both 6 day-treated (*n* = 3) and control (*n* = 3) groups

PET monitoring was only performed for set A of mice. In the reconstructed images, the signal appeared more prominent in the tumour, bladder and heart compared to the rest of the body (Fig. 2a). At randomization, [^18^F]FDG uptakes expressed in SUV were similar in both groups. In the control group, the tumour [^18^F]FDG uptake was 1.4 greater after 5 days than at randomization, from 1.2 ± 0.1 to 1.6 ± 0.3 (*n* = 3) in SUV, although the difference was not statistically significant (NS). In the treated group, the [^18^F]FDG uptake remained stable during sunitinib administration, from 1.1 ± 0.2 to 1.1 ± 0.2 (*n* = 4) in SUV (NS). As a result, after 5 days of treatment, the [^18^F]FDG uptake was significantly lower, by a factor of 1.5 (*p* < 0.05), in the treated versus the control tumours (Fig. 2b).

The [^18^F]FMISO PET images exhibited an enhanced contrast in the tumour and intestine regions (Fig. 2c). At randomization, [^18^F]FMISO tumour uptake was not significantly different between the group to be treated (1.2 ± 0.2 in SUV, *n* = 3) and the control group (0.8 ± 0.1 in SUV, *n* = 3), although lower in the control group. After 6 days of sunitinib or vehicle only administration, the tracer uptake remained stable in the control group (0.9 ± 0.2 in SUV, *n* = 3) and decreased in the treated one (0.9 ± 0.4 in SUV, *n* = 3), thus reducing the initial differences between the two groups (Fig. 2d).

### *Ex vivo* evaluation of sunitinib incidence on PyMT tumour hallmarks

**Fig. 3:**
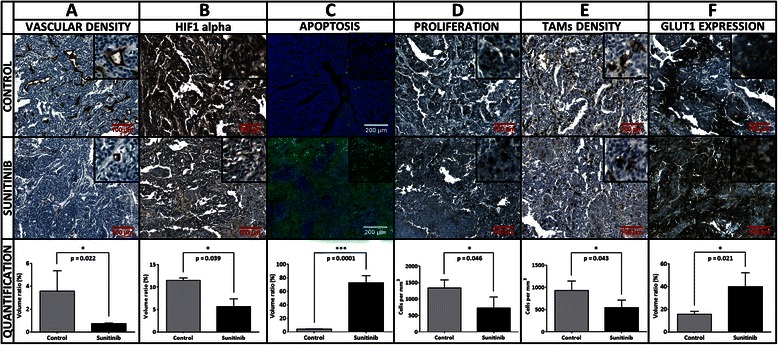
Biomarker quantification by digital microscopy. Each column corresponds to a labelling: **a** CD31, **b** HIF1 alpha, **c** TUNEL, **d** Ki-67, **e** F4/80, **f** GLUT1. Representative control and 9 day-treated tissues are displayed on first and second rows respectively. The third row presents the associated values. In bright field images, the biomarker of interest is labelled in brown and nuclei are counterstained in blue. In fluorescence images, TUNEL labelling is represented in green and nuclei are counterstained in blue

**Fig. 4:**
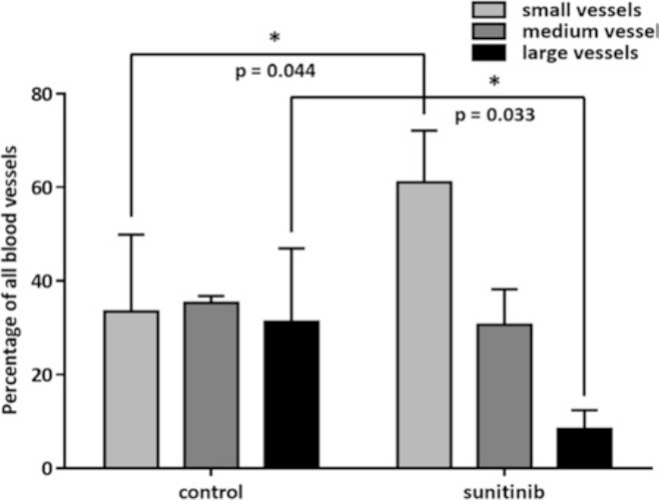
Sunitinib effect on blood vessel size. Comparison of the proportion of small, medium and large vessels between the control and treated groups (set A of mice)

**Fig. 5:**
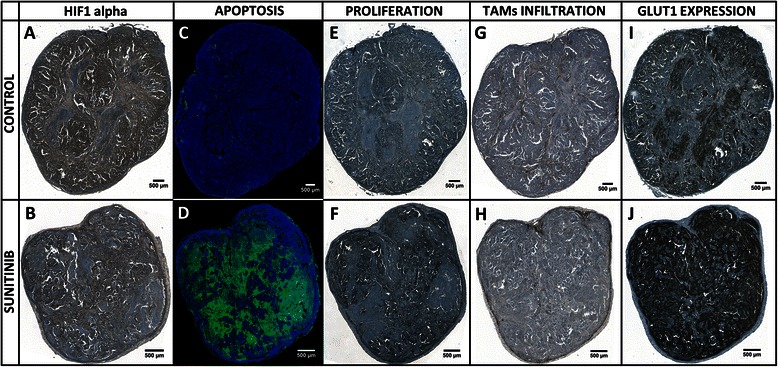
Whole tumour slide imaging. HIF1 alpha: (**a**) in control tumours, the HIF1alpha expression is mainly located inside heaps of high cellularity (**b**) in treated ones, the labelling is weaker, globally as necrotic regions get larger, and even locally inside living cell islets. TUNEL: (**c**) control section with low level of apoptosis (**d**) highly apoptotic sunitinib-treated tumour. Ki-67: (**e**) proliferating cell density remains at a relatively low level in control tumours, whereas (**f**) in treated ones, necropsied areas get larger but the density of Ki-67 positive cells increases in the remaining living cell islets. F4/80: (**g**) in controls, the highest TAMs density is encountered at the interface of tumour and necrotic regions; (**h**) in treated tumours, TAMs tend to relocate at the tumour external edges. GLUT1: (**i**) in control conditions, necrotic areas are the place of high GLUT1 expression; (**j**) under sunitinib treatment, necropsied areas are larger and GLUT1 expression changed accordingly

Digital microscopy analysis was exclusively performed on set A of mice. In the control and treated groups, the mean hematoxylin volumes estimated using digital microscopy were 126 ± 6 (*n* = 3) and 45 ± 20 mm^3^ (*n* = 4) respectively (*p* < 0.01). The nine day sunitinib treatment induced a 4.9 fold regression of the vascular volume reported to the hematoxylin volume, with values of 3.6 ± 1.8 % (*n* = 3) and 0.7 ± 0.05 % (*n* = 4) in the control and treated groups respectively (*p* < 0.05) (Fig. 3a). The sunitinib treatment induced a reduction of the large vessel proportion (from 31.3 ± 15.6 % in the control group to 8.3 ± 4.0 % in the treated group, *p* < 0.05), an increase of the small size vessels (from 33.4 ± 16.5 % in the control group to 61.0 ± 11.1 % in the treated group, *p* < 0.05) and no evolution for medium size vessels (Fig. 4). Interestingly, the vascular volume decrease did not induce a global increase in HIF1 alpha protein expression (Figs. 3b and 5a-b). On the contrary, sunitinib therapy led to a twofold reduction of HIF1 alpha labelling, from 11.5 ± 1.0 % (*n* = 3) in control tumours to 5.7 ± 3.5 % in treated ones (*n* = 4, *p* < 0.05). TUNEL labelling revealed a high induction of apoptosis by a factor of 17.7. Indeed, in nutrient supplied expanding tumours, very low programmed cell death was observed, representing 4.1 ± 0.3 % in volume (*n* = 3), while this value was 72.6 ± 10.7 % in treated tumours (*n* = 4, *p* < 0.001) (Figs. 3c and 5c-d). A mean pool of 1339 ± 248 cells per mm^3^ of tumour (*n* = 3) were over-expressing Ki-67 in non-treated tumours and the therapeutic agent reduced this population to 730 ± 334 proliferating cells per mm^3^ (*n* = 4, *p* < 0.05). This represents a 1.8 fold reduction of the tumour proliferation process (Figs. 3d and 5e-f). Tumours grown in the control conditions presented a mean density of 933 ± 212 macrophage cells (F4/80 positive) per mm^3^ of viable tumour tissue (*n* = 3). In the sunitinib treated mice, this value was at 546 ± 169 macrophages per mm^3^ (*n* = 4) (Figs. 3e and 5g-h) (*p* < 0.05 compared to the control mice). Finally, GLUT1 whole tumour expression was enhanced by a factor of 2.57 in the sunitinib treated group when compared to control (Figs. 3f and 5i-j). Indeed, transporter labelling represented 15.5 ± 2.6 % of control hematoxylin volume (*n* = 3), and reached 40.0 ± 12.3 % after the 9 days-long treatment (*n* = 4, *p* < 0.05).

### Impact of sunitinib administration on the metastatic dissemination in lungs

**Fig. 6:**
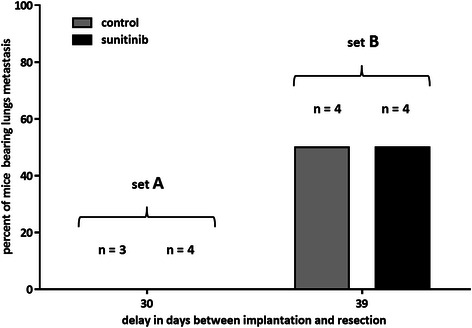
Lung metastasis incidence according to the primary tumour growth duration in control and treated groups. The percentage of lungs bearing metastasis is plotted against the delay between tumour implantation and resection. At 30 days, no lung metastasis is present in treated (*n* = 4) and control (*n* = 3) groups (set A). At 39 days, metastases are detected in half the lungs in both the 14 day-sunitinib treated group (*n* = 4) and the corresponding control group (*n* = 4) (set B)

In set A, with a primary tumour growth duration of 30 days, comprising a 9-day sunitinib or DMSO administration, no lung metastasis was detected 60 days after tumour resection in the treated (*n* = 4) and control (*n* = 3) groups. In set B with a 39 days tumour growth duration, including 14 days of sunitinib or DMSO treatment, the incidence of lung metastasis was of 50 % in the treated (*n* = 4) and control (*n* = 4) groups (Fig. 6).

## Discussion

With an ER+/− PR+/− HER2+ status and a luminal transcriptomic signature, the PyMT model is considered to mimic human luminal B (HER2-positive) breast cancers [23–25]. The monitoring of mouse body weights during the sunitinib administration phases revealed no significant variation by comparison to control groups, which suggests that the drug, when administered at 40 mg/kg per os, had acceptable toxicity on this model. The 9-day sunitinib treatment induced a significant regression of the tumour volume and a 14 day-treatment duration further reduced the tumour volumes when compared to their respective controls. These results are consistent with those reported by Bousquet [13] and Abrams [15] regarding the luminal mammary cancer responsiveness to sunitinib.

The high efficiency of sunitinib on the MMTV-PyMT model by comparison to TNBC models might be partly explained by its dependency to particular pathways activated by the middle-T oncogene. Indeed, the middle-T antigen was shown to act through co-optation of several transduction pathways including: i) the protein phosphatase 2A (PP2A) activating the cytosolic tyrosine kinases (PTK) of the *Src* family (Src, Fyn, Yes), ii) the phospholinositide 3-kinase (PI3K) activating the Akt/mTOR cell-survival pathway, iii) the mitogen-activated protein kinases (MAPK/ERK) pathway through recruitment of the Shc adapter protein and iv) the phospholipase Cγ1 (PLC-γ) pathway inducing the protein kinase C (PKC) activation and a cytosolic Ca^2+^ concentration increase [26, 27]. As highlighted in Additional file 3, sunitinib is known to interact with the *Src* family cytosolic tyrosine kinases and with more than 10 tyrosine kinase membrane receptors that participate in the regulation of the MAPK/ERK and PI3 kinase pathways. As recently demonstrated on a medulloblastoma model, this drug might also repress these two signalling cascades through the induction of PTEN expression [28].

In addition to the decrease in tumour size, the vascular network evaluation demonstrated that sunitinib impacts vascular density and maturity. Moreover, the treated tumours were characterised by a higher level of apoptosis by comparison to controls. Previously, this multi-kinase inhibitor has already been shown to present *in vitro* and *in vivo* anti-angiogenic effects as well as direct pro-apoptotic properties [29, 30]. Regarding TAMs, their density was slightly reduced under sunitinib treatment versus control. As recently reviewed, TAMs roles are many and include the promotion of neo-angiogenesis, tumour immune evasion and metastatic behaviour [31]. To our knowledge our study is the first to evaluate the therapy response to sunitinib of primary tumours in a mammary cancer model using [^18^F]FDG or [^18^F]FMISO PET. The closest related work describes a [^18^F]FDG PET monitoring of the sunitinib response on lung metastases in a 4T1 intravenously induced metastatic model [32] and showed an increased [^18^F]FDG signal in the lungs of the sunitinib-treated mice compared to the control mice, which correlated with an enhanced seeding of lung metastases associated with sunitinib administration. In our [^18^F]FDG PET data, the mean SUV increased during the 5 day-tumour growth in the control group, whereas it remained stable in the treated tumours. We checked that the stable [^18^F]FDG uptake in treated tumours that were concomitantly decreasing in size was not due to partial volume effect (PVE) [33] and found that PVE alone could not explain our observations. The uptake mechanism of [^18^F]FDG has been previously studied emphasizing the role of the GLUT protein family [34]. In our work, we only measured GLUT1 expression and showed that sunitinib increased the presence of this transporter. The associated lack of increase in apparent [^18^F]FDG uptake in sunitinib-treated tumours might be at least partly explained by the lower levels in vascularisation, TAM infiltration and cell viability in sunitinib treated by comparison to control tumours. Yet, the overall conclusion is therefore that [^18^F]FDG PET evidenced the response to sunitinib treatment in this tumour model.

In our [^18^F]FMISO PET scans, randomization did not yield two perfectly equivalent groups regarding the hypoxia levels as expressed in SUV. Nevertheless, the untreated tumours remained stable in hypoxia over the treatment course, whereas the sunitinib administration tended to reduce hypoxia, although the difference was not significant in our small sample. Therefore, despite the reduction in blood supply, the treated tumours did not become more hypoxic than before the sunitinib administration. This might seem paradoxical as the sunitinib-induced anti-angiogenic effects are often associated with an increase in hypoxia due to the tumour starvation in nutrients. This enhanced hypoxia phenomenon has for instance been described by Welti et al. [32] and contributes to explain the sunitinib efficacy on the preclinical models. In our case, even in absence of enhanced hypoxia, we observed a huge increase of the apoptotic level in the treated tumours compared to the control ones. As explained above, the sunitinib is known to repress many cell survival pathways that are over-activated by the middle-T oncoprotein, and to present pro-apoptotic properties on tumour cells [30]. This supports the idea that the tumour cell apoptosis observed in our model might be mainly induced by the direct pro-apoptotic properties of sunitinib, owing to its multi-kinase inhibitor activity. Indeed, since more apoptosis occurred in the sunitinib-treated tumours compared to the control ones, the drug induced tumour regression, which finally could explain the absence of enhanced hypoxia even in a reduced angiogenesis context. The HIF1 alpha protein has a central role in the cellular adaptation process under a stressful hypoxic environment. Its regulation has been extensively reviewed [35]. Here the mild, but not statistically significant, decrease of tumour hypoxia observed in the sunitinib group was concomitant with a reduced level of HIF1 alpha expression in sunitinib tumours compared to control ones. [^18^F]FMISO PET therefore appeared useful to characterise the hypoxia level inside the tumours, and also to unveil the preferential way of action of the drug on this model.

Interestingly, under sunitinib treatment, apoptosis was highly increased by a factor of 17.7 whereas Ki-67-positive cell number decreased only by a factor of 1.8 when compared with the control group. Areas of proliferating cells were reduced but the Ki-67 marker was denser in the remaining living cell islets. Thus, we hypothesize that a resistance mechanism of a few cancer cells to sunitinib might act through an induction of their cell-division cycle. Moreover, two recent publications proved that one of the effects of this drug on TNBC xenograft models is to increase the cancer stem cells (CSCs) population [36] by generating intra-tumoral hypoxia [37]. Further investigations might tell whether with no increased hypoxic level, as observed in our model, the proliferating cell pool still displays a few typical CSCs markers. Those cells are indeed of major importance as they present enhanced epithelial-mesenchymal transition properties and thus high metastatic potential [38]. Their promotion under sunitinib treatment might at least partly explain its disappointing efficiency on several models of metastasis [32, 39]. In our work, the 9 day-treated set A of mice did not allow us to study the effect of the drug on the metastatic process. Comparing the 14 day-treated group against controls (set B) implanted for 39 days, the treatment did not appear to impact the incidence of lung metastases. Further molecular characterisation of this sunitinib-resistant cellular pool is required to specifically target them, for instance by combining sunitinib treatment with a c-Met inhibition strategy using crizotinib [40].

## Conclusion

We showed that the luminal B (HER2-positive) type PyMT model was particularly sensitive to sunitinib compared to other preclinical breast cancer models, such as TNBC models that have been extensively used to study the effects of this drug. Our histology, [^18^F]FDG PET and [^18^F]FMISO PET imaging results suggest that in addition to its anti-angiogenic effects, the sunitinib efficacy on this model is mostly due to its direct pro-apoptotic properties.

## Additional files

Additional file 1: **General diagram for the CellProfiler pipeline dedicated to image segmentation of GLUT1, HIF1 alpha, KI67 and F4/80 labelled tissue slides.** This example displays the step-by-step image processing of a tumour tissue labelled for the F4/80 antigen. The modules used in the pipeline are noted in bold. The image names appear in italic by the image side.
Additional file 2: **CellProfiler pipeline for vessel segmentation and clustering in CD31 labelled tissue slides.** The above example displays the step-by-step image processing of a tumour tissue labelled for the CD31 antigen. The modules used in the pipeline are noted in bold. The image names appear in italic by the image side.
Additional file 3: **List of the main known high affinity targets for sunitinib.** Sunitinib interaction partners were determined using a semi-quantitative affinity chromatography method followed by LC/MS analysis. Data collected from Bairlein et al. [29].

## Abbreviations

[^18^F]FDG: 2-deoxy-2-[^18^F]fluoro-D-glucose
[^18^F]FMISO: [^18^F]fluoromisonidazole
%ID/g: Percent of injected dose per gram
2D-OSEM: Two dimensional ordered-subset expectation maximization
CSCs: Cancer stem cells
DAB: 3-3'–diamino-benzidine
DAPI: 4',6'-diamidino-2-phenylindole
DMSO: Dimethyl sulfoxyde
ER: Estrogen receptor
GLUT1: Glucose transporter 1
HER2: Human epidermal growth factor receptor 2
HIF1 alpha: Hypoxia induced factor 1 alpha
MMTV: Mouse mammary tumour virus
NS: Not significant
ORR: Overall response rate
PET: Positron emission tomography
PMID: Pubmed-indexed for MEDLINE
PR: Progesterone receptor
PVE: Partial volume effect
PyMT: Polyoma virus middle T
qRT-PCR: Quantitative real time polymerase chain reaction
RNA: Ribonucleic acid
ROIs: Regions of interest
SD: Standard deviation
SUV: Standardized uptake value
TAMs: Tumour-associated macrophages
TNBC: Triple negative breast cancer
TSA: Tyramide signal amplification
TUNEL: Terminal deoxynucleotidyl transferase dUTP nick end labelling
TZM: Trastuzumab
