# Supplementary figures and images for: Monitoring therapeutic efficacy of sunitinib using [18F]FDG and [18F]FMISO PET in an immunocompetent model of luminal B (HER2-positive)-type mammary carcinoma
Source: BMC Cancer. 2015 Jul 22;15:534. doi: 10.1186/s12885-015-1540-2 (PMC4511439; doi:10.1186/s12885-015-1540-2)

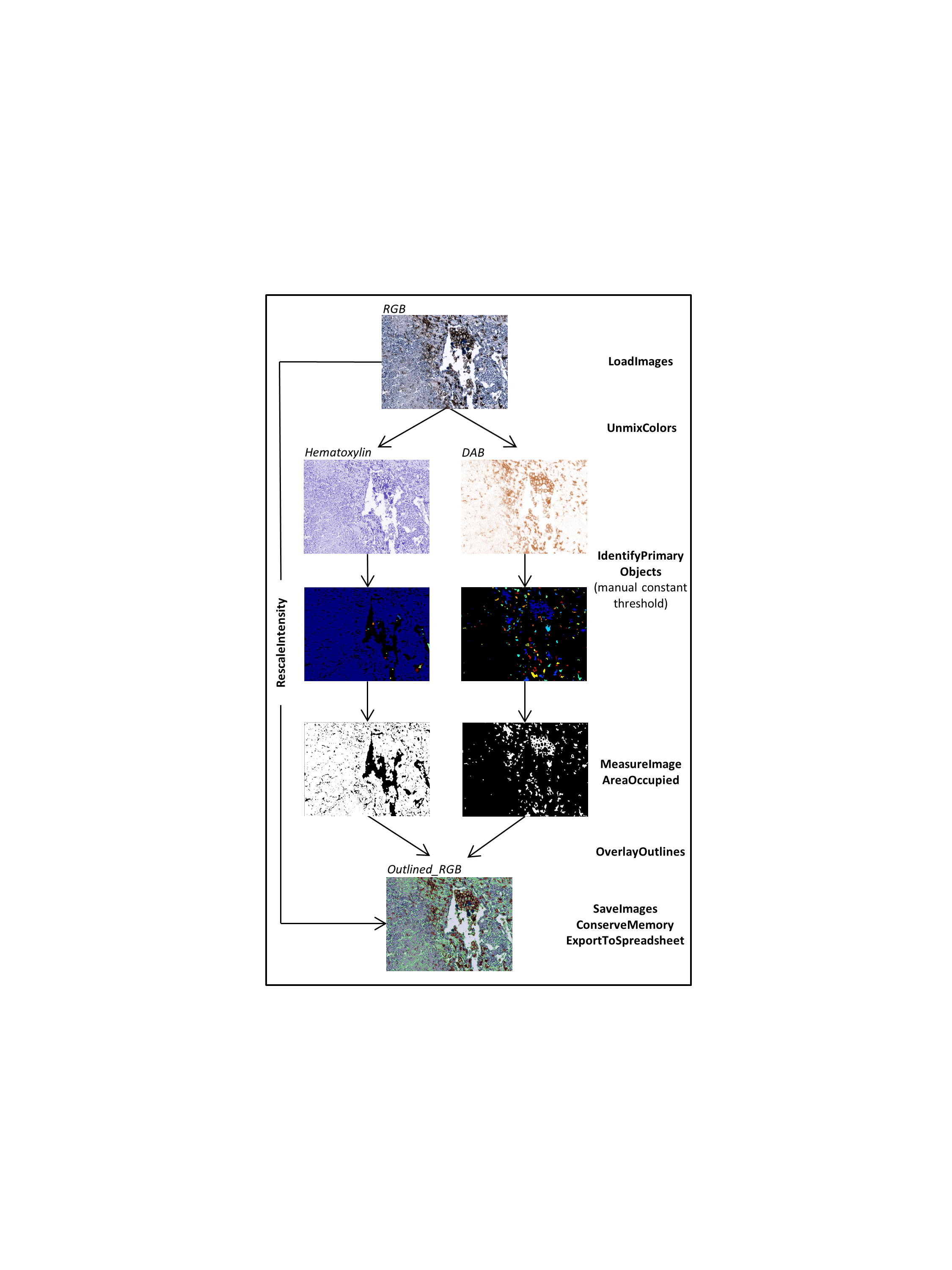

Supplement: Additional file 1: — General diagram for the CellProfiler pipeline dedicated to image segmentation of GLUT1, HIF1 alpha, KI67 and F4/80 labelled tissue slides. This example displays the step-by-step image processing of a tumour tissue labelled for the F4/80 antigen. The modules used in the pipeline are noted in bold. The image names appear in italic by the image side. [file 12885_2015_1540_MOESM1_ESM.tiff]

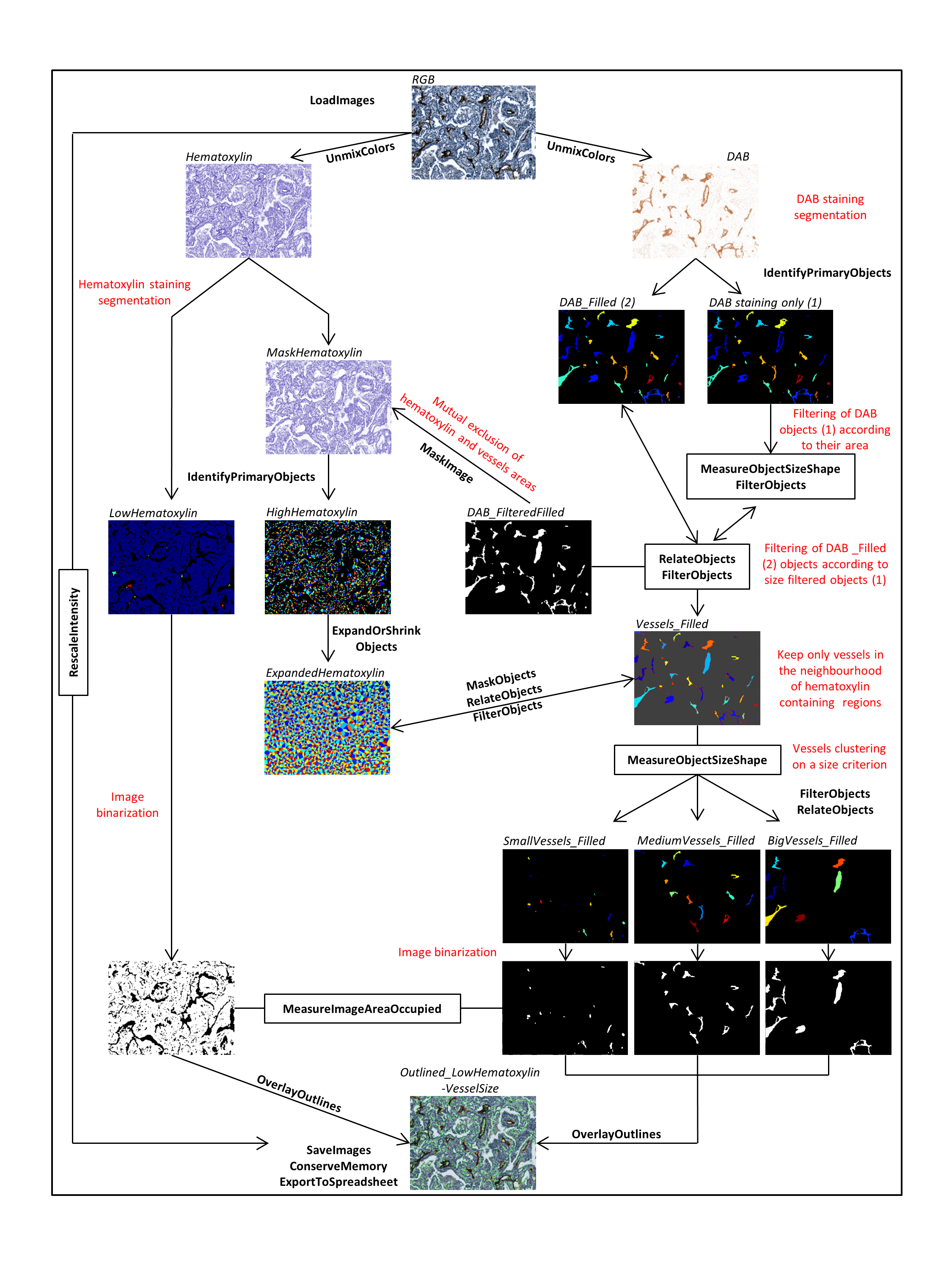

Supplement: Additional file 2: — CellProfiler pipeline for vessel segmentation and clustering in CD31 labelled tissue slides. The above example displays the step-by-step image processing of a tumour tissue labelled for the CD31 antigen. The modules used in the pipeline are noted in bold. The image names appear in italic by the image side. [file 12885_2015_1540_MOESM2_ESM.tiff]
