# Supplementary material for: Monitoring therapeutic efficacy of sunitinib using [18F]FDG and [18F]FMISO PET in an immunocompetent model of luminal B (HER2-positive)-type mammary carcinoma
Source: BMC Cancer. 2015 Jul 22;15:534. doi: 10.1186/s12885-015-1540-2 (PMC4511439; doi:10.1186/s12885-015-1540-2)
Supplement: Additional file 3: — List of the main known high affinity targets for sunitinib. Sunitinib interaction partners were determined using a semi-quantitative affinity chromatography method followed by LC/MS analysis. Data collected from Bairlein et al. [29]. [file 12885_2015_1540_MOESM3_ESM.pdf]

Protein families	HGNC gene coding symbol	Protein description
**Tyrosine kinase receptors**	**PDGFRA**	platelet-derived growth factor receptor, alpha polypeptide
	**PDGFRB**	platelet-derived growth factor receptor, beta polypeptide
	**VEGFR1-2-3**	vascular endothelial growth factor receptor 1,2,3
	**FLT3**	fms-related tyrosine kinase 3
	**KIT**	v-kit Hardy-Zuckerman 4 feline sarcoma viral oncogene homolog
	**RET**	glial cell-line derived neurotrophic factor receptor
	**CSF1R**	colony stimulating factor 1 receptor
	**AXL**	AXL receptor tyrosine kinase
	**TYRO3 (SKY)**	TYRO3 protein tyrosine kinase
	**MERTK**	c-mer proto-oncogene tyrosine kinase
	**FGFR1**	fibroblast growth factor receptor 1
	**MST1R (RON)**	macrophage stimulating 1 receptor (c-met-related tyrosine kinase)
	**MET**	*met* proto-oncogene (hepatocyte growth factor receptor)
	**ROS1**	c-ros oncogene 1, receptor tyrosine kinase
**Cytosolic tyrosine kinases**	**PTK2 (FAK)**	PTK2 protein tyrosine kinase 2
	**FER**	*fer* (fps/fes related) tyrosine kinase
	**LYN**	v-yes-1 Yamaguchi sarcoma viral related oncogene homolog
	**FYN**	*FYN* oncogene related to SRC, FGR, YES
	**JAK1**	Janus kinase 1
	**YES1**	v-yes-1 Yamaguchi sarcoma viral oncogene homolog 1
	**SRC**	v-*src* sarcoma (Schmidt-Ruppin A-2) viral oncogene homolog (avian)
**Non-tyrosine kinases**	**NEK9**	NIMA (never in mitosis gene a)- related kinase 9
	**BMP2K**	BMP2 inducible kinase
	**TBK1**	TANK-binding kinase 1
	**AAK1**	AP2 associated kinase 1
	**RPS6KA1**	ribosomal protein S6 kinase, 90kDa, polypeptide 1
**Non-kinase proteins**	**NME4**	NME/NM23 nucleoside diphosphate kinase 4
	**NQO2**	ribosyldihydronicotinamide dehydrogenase
	**ACOT7**	glycogen phosphorylases, the Acyl-CoA thioesterase 7
	**NQO1**	NAD(P)H dehydrogenase 1
	**ENO1**	alpha-enolase
	**ENO3**	beta-enolase
